# Arab representation in Israeli healthcare professions: achievements, challenges and opportunities

**DOI:** 10.1186/s13584-024-00663-3

**Published:** 2025-02-03

**Authors:** Bruce Rosen, Sami Miaari

**Affiliations:** 1 Myers-JDC-Brookdale Institute, Jerusalem, Israel; 2 The Hebrew University, Jerusalem, Israel; 3 Tel Aviv University, Tel Aviv, Israel; 4 Yale University, New Haven, CT USA

**Keywords:** Health care professions, Professional studies, Arabs in Israel

## Abstract

**Background:**

Israel is a multiethnic society with a population of 9.8 million at the end of 2023. Israeli Arabs (i.e., Arab Palestinian citizens of Israel) account for 21% of the Israel’s overall population, 22% of its working age population and 16% of the employed population. This study has several objectives: (1) To provide an overview of the current representation of Israeli Arabs in four key health care professions (medicine, nursing, dentistry, and pharmacy) in terms of employment, licensure, and professional studies; (2) To document changes in those parameters over the past decade, subject to limitations of data availability; (3) To provide a broader context on the employment of Israeli Arabs; (4) To explore the policy implications of the key findings.

**Methods:**

Estimates of employment levels are based on the Labor Force Survey (LFS) of the Central Bureau of Statistics (CBS). Data on licensure (i.e., professional qualification) and place of professional studies were extracted from the Ministry of Health report series entitled “The Health Care Professions”. Data on enrollment in degree programs in Israel was provided by the Council of Higher Education. Important background information was elicited from relevant policy documents and policy experts.

**Results:**

In 2023, among employed Israelis up to age 67, Arabs constituted approximately one-quarter of Israel’s physicians (25%), nurses (27%), and dentists (27%), and half of Israel’s pharmacists (49%). These percentages are substantially higher than they were in 2010, with the increase being particularly marked in the case of physicians (25% versus 8%). The number of new licenses granted annually increased significantly between 2010 and 2022 for both Arabs and Jews in each of the professions covered. The percentage of newly licensed professionals who are Arab increased substantially among physicians and nurses, while remaining stable among dentists and pharmacists. In medicine, dentistry, and pharmacy, many of the licensed Arab health professionals had studied outside of Israel; this phenomenon also exists for nursing but is less widespread there. In the 2022/3 academic year, the percentage of first-degree students in Israeli colleges and universities who were Arab was 70% in pharmacy, 33% in nursing, 23% in dentistry, and 9% in medicine. Between 2012/3 and 2022/3 the percentage of first-degree students who are Arab increased substantially for pharmacy, declined slightly for nursing, and declined substantially for medicine and dentistry.

**Conclusions:**

Arab professionals play a substantial and recently increased role in the provision of health care services in Israel. It is important to recognize, appreciate, and maintain this substantial role. Moreover, its potential as a model for sectors other than health care should be explored. To build on achievements to date, and to promote continued progress, policymakers should expand access to health professional education within Israel, upgrade the skills of graduates of non-Israeli universities, promote diversity in leadership positions and key specialties, and expand specialty care services in Arab localities.

**Supplementary Information:**

The online version contains supplementary material available at 10.1186/s13584-024-00663-3.

## Background

The issue of diversity in the healthcare workforce has received substantial attention in both national policy documents and in the international scholarly literature. Countries which have given substantial attention to this issue include the United States [1–5], the United Kingdom [6–9], Holland [10, 11], Germany [12, 13], and Israel [14–16]. However, there have been no recent empirical, English language, articles about the representation of minorities in the health professions in Israel.

The international literature includes both conceptual articles about why representation of minorities and diversity are important and empirical articles about actual levels of representation (See Appendix A). The literature reflects an understanding that adequate representation and advancement of minorities is important for both minority patients (in terms of access to high-quality, culturally responsive, care) and minority workers (in terms of economic and social mobility) [6, 17].

Almost all of the empirical articles are country specific.^1^ Most of them also focus on medicine, though there is some attention to other professions. One of most highly cited empirical articles in this literature [1] examined the prevalence of underrepresented minorities in a broad range of health professions (including medicine, dentistry, nursing, and pharmacy) in the United States. It considered both the stock of employed professionals (reflecting current minority representation) and the pipeline of students in professional schools (as an indication of potential changes in representation). Strikingly, that article found that Black, Hispanic, and Native Americans are seriously underrepresented in almost all the health professions covered.

There are several reasons why the current situation in Israel should be of particular interest to scholars, practitioners, and policymakers in other countries. First, while in many countries it is often difficult to disentangle minority status from immigration status, in Israel almost all the minority health professional are native born. Second, the Israeli setting provides an opportunity to learn about minority representation in a context where many members of the main minority group have ethnic and other links with residents of adjacent jurisdictions, some of which are not at peace with their home country.

Israel is a multiethnic society with a population of 9.8 million at the end of 2023 [19]. In 2022, Israeli Arabs constituted 21% of the overall population of the State of Israel and 22% of its working age population. Historically, this demographic group has faced numerous socioeconomic challenges, including lower levels of education, employment and income levels than their Jewish counterparts [20, 21].^2^

However, the socio-economic situation of Israeli Arabs has improved in recent decades [22–24], as the increase in the prevalence of higher education has helped many Arab families move from the lower class to the middle class. For example, the number of Israeli Arabs pursuing higher education more than doubled from 25,951 in 2009–2010 to 57,552 in 2020–2022 [25]. There has also been a major increase in the number of Israeli Arabs securing employment in professional or academic positions [26].

The health care professions have been, and continue to be, an important vehicle for social and economic mobility for Israel’s Arab population, offering pathways to white collar employment and social integration [27]. For many Israeli Arabs, study outside of Israel has been an important pathway to licensure as a health care professional [15, 28, 29].

Aside from the health professions, the main profession in which Israeli Arabs have been prominent is teaching in primary and secondary schools. This has been an important vehicle for social mobility of Arab professionals and for the education of Arab students. It differs from the health professions in that employment is predominantly within the Arab enclave, wage levels are relatively low, and demand has been largely saturated [30, 31].

Many Israeli Arabs are also employed in other non-health professions such as law, accounting and engineering. However, their representation and prominence in these professions is less than in the health care professions. Moreover, in law and accounting many of the Arab professionals are self-employed within the Arab enclave, in part to avoid unfair practices of the large Jewish firms^3^ [32]. To our understanding very few scholarly articles have been written about the presence of Israeli Arabs in professions outside of health care.

In contrast, much is already known about the representation and experiences (both positive and negative) of Israeli Arabs in the fields of medicine [16, 33, 34], nursing [33, 35–37], and pharmacy [38, 39], as well as the health professions more generally [16]. The motivation of Israeli Arabs to enter these fields has also been explored [36, 40] as have the benefits of a diverse workforce to patients and to the health care system overall [34, 37, 41]. In addition, Israel’s Ministry of Health publishes an annual report about the health care workforce, indicating the share of Israeli Arabs among newly licensed professionals, and among all working age licensees, in each of the major health care professions [42]. Many of the developments that have taken place in Israel are in line with broader, global trends that recognize the importance of minority representation in critical sectors and the benefits of a diverse workforce in healthcare delivery [34].

The theoretical frameworks underpinning studies of Israeli Arabs in the health professions draw on intersectionality theory, examining how multiple identities—ethnicity, gender, religion, and profession—interact in shaping career trajectories and workplace experiences [16]. Additionally, cultural competence models and structural competence models have been employed to understand the impact of workforce diversity on healthcare delivery [43, 44].

Despite the growing body of research on the roles and experiences of Israeli Arabs in the health care professions, a comprehensive overview of recent trends in the employment, licensure (i.e., qualification), and education of Israeli Arabs in a set of major healthcare professions remains conspicuously absent from the literature. This gap is particularly significant given the rapid changes in the educational and professional landscapes over the past decade. The present study aims to bridge this gap by providing an integrated overview of Israeli Arab participation across a set of four major health professions.

Israel’s Ministry of Health regulates approximately twenty professions. Four of these—medicine, dentistry, nursing, and pharmacy—are both large (i.e., over 5,000 licensed professionals of working age)^4^ and characterized by a high percentage of Arabs among licensed professionals of working age (i.e., over 20%) [42]. Those four professions are the focus of this article. An important contrast is provided by another large health profession—psychology—in which Arabs account for a relatively low percentage of licensed professionals (7%). Appendix B provides additional information on the role of Israeli Arabs in psychology.^5^

Most articles about the health care professions in Israel tend to focus on a single profession such as nursing or medicine.^6^ We acknowledge that a focus on a particular profession can provide more depth than is possible in an article—such as this one—that covers numerous professions. At the same time, we believe that there is also value in providing information jointly on several major health professions: Professionals in these fields are dedicated to addressing a common human need, health; are overseen by the same government agency (the Ministry of Health); and tend to be employed by the same set of hospitals and health plans. Multi-professional articles such as this one make it possible to identify similarities and differences across professions.

**Fig. 1:**
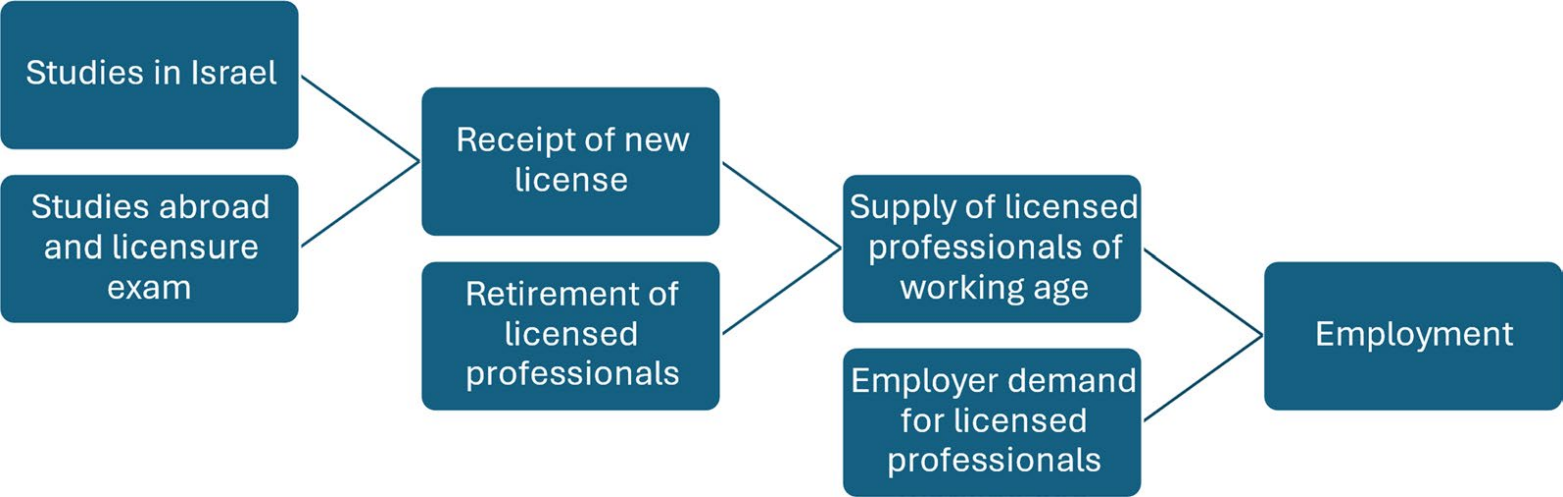
Flowchart of professional studies, licensure, and employment

While many articles about health care professionals in Israel focus either on employment, licensure, or professional education, this article takes an integrated approach to those elements, as depicted in Fig. 1.

The diagram illustrates three points not always emphasized in the professional literature:

1. Employment is influenced not only by preferences and supply of potential employees but also by the preferences and demand of potential employers.
2. The supply of employees is influenced not only by inflow (i.e., receipt of new licenses) but also by outflow (retirement, etc.).
3. The inflow of newly licensed professionals is influenced not only by professional studies in Israel but also by professional studies abroad.

As will be detailed below, the availability of data on these parameters vary: for some no data are available; for others data are available but without differentiating between Arabs and Jews; and for still others data differentiating between Arabs and Jews are available. In any case, it is important to keep in mind the full set of parameters—both to understand the substance and limitations of the present study and to lay the groundwork for expansions of the present study.

Several major governmental planning and programmatic initiatives, which are either underway or have recently been completed, have significant implications for the participation of Israeli Arabs in the health care professions. These include:

1. A set of 5-year national plans to improve the economic and social wellbeing of Israeli Arabs through the infusion of substantial new funds, involvement of many government ministries, and the establishment of a special unit to coordinate these efforts [46, 47]
2. A Council of Higher Education initiative to substantially increase the number of Israeli Arabs in Israeli colleges and universities and increase their diversification across departments [25, 48, 49]. Key components of this initiative include enhanced exposure to academia during high school; preparatory programs/schools to increase the likelihood of acceptance and success in higher education by upgrading language and other skills; and post-matriculation assistance such as financial support, financial incentives to study in departments particularly relevant to the Israeli job market,^7^ educational and professional guidance, and emotional/social support.
3. An inter-ministerial initiative to improve the absorption in employment in Israel of Israeli Arabs who studied in other countries [15]. Key recommendations include expanding study opportunities in Israel, discouraging studies at non-Israeli institutions of lesser quality, facilitating study at non-Israeli institutions of higher quality, and providing supplemental training in Israel to upgrade skill levels.
4. A Ministry of Health planning effort to reduce inequalities in health care, including special attention to the Arab population [50]. Efforts underway include the establishment of health units in Arab localities and investment in infrastructures and programs to promote healthy lifestyles.
5. The establishment within the Ministry of Health of a new unit focused on workforce planning and a related increase in analysis and planning efforts [51]. These include several initiatives to increase the number of new physicians from, and in, the periphery (where most Israeli Arabs live) and to ease the absorption of Israelis who studied medicine abroad (most of whom are Israeli Arabs).

Reports from these planning efforts have informed this article and it is our hope that this article will in turn inform those planning efforts and subsequent programs.

This study has several objectives:

1. To provide an overview of the current representation of Israeli Arabs in four key health care professions (medicine, nursing, dentistry, and pharmacy) in terms of employment, licensure, and professional studies
2. To document changes in those parameters over the past decade, subject to limitations of data availability.
3. To contextualize healthcare’s role in Arab socio-economic mobility by comparing Arab representation in the healthcare professions to Arab representation in other professions and occupations.
4. To highlight policy initiatives that can preserve Arab representation in the health care professions and enhance inclusion in leadership roles.

The study is grounded in three interrelated theoretical frameworks that collectively help explain the representation of Israeli Arabs in the healthcare professions: social mobility theory [52], cultural competence theory [53], and (to a lesser extent) intersectionality theory [54]. Each provides a lens to understand the dynamics of Arab participation, challenges, and opportunities in healthcare professions and to develop polices for creation of an optimal heath care workforce. Further information on these theories can be found in Appendix C.

## Methods

The overview considers both point-in-time “stocks” (for employment and licensed professionals) and annual “flows” (for newly licensed professionals and recent professional studies). In particular, the analysis of licensure data considers both stocks (the total number of licensed professionals at any point in time, irrespective of when they received their licenses) and flows (the number of newly licensed professionals in each year). While the stock of licensed professionals is affected both by inflow (newly licensed professionals) and outflow (retirees, etc.), this overview presents data only on the inflow, as data on the outflow were not readily available.

Regarding professional studies, this overview considers both studies in Israel and studies abroad. However, regarding studies abroad, data on the Arab–Jewish mix are available only for medicine.

Estimates of employment levels are based on the Central Bureau of Statistics’ Labor Force Survey. Data on licensure and place of professional studies were extracted from the Ministry of Health (MOH) report series entitled “The Health Care Professional Workforce” [42]. Data on enrollment in degree programs in Israel was provided by the Council of Higher Education (CHE). Important background information was elicited from relevant policy documents [15, 48, 55] and interviews with several health policy experts.^8^

All statistics include Arab Palestinian citizens of Israel and Palestinians from East Jerusalem because the data from major administrative sources do not differentiate between these regions. It does not include Palestinians from the West Bank and Gaza.

In keeping with standard Central Bureau of Statistics (CBS) practice, the variable “population group” is divided into two groups: “Arabs” and “Jews and others”. As the latter group is predominantly composed of Jews, we refer to it as “Jews” throughout this article.

All the data sets used are very reliable and all use the same definition of ethnicity. Differences between related variables in percentage changes over time are to be expected because they are nonetheless distinct variables. For example, the percentage change over time for licensure can be different from the percentage change for studies at Israeli institutions of higher learning due to at least two factors: many licensed professionals studied at non-Israeli institutions and not everyone who studies at an institution of higher learning completes the course of study and passes the relevant licensure examination. Similarly, licensure and employment are related but distinct; not all licensed professionals choose to work in any given year, and not all those who seek to work succeed in finding employment.

The analysis of the Labor Force Survey consisted of point-in-time comparisons between Arabs and Jews for the years 2010 and 2022, as well as analyses of changes between those years for each population group. Unless otherwise noted, all differences between groups and changes over time that are mentioned in the text were statistically significant.

## Results

### Employment

**Fig. 2:**
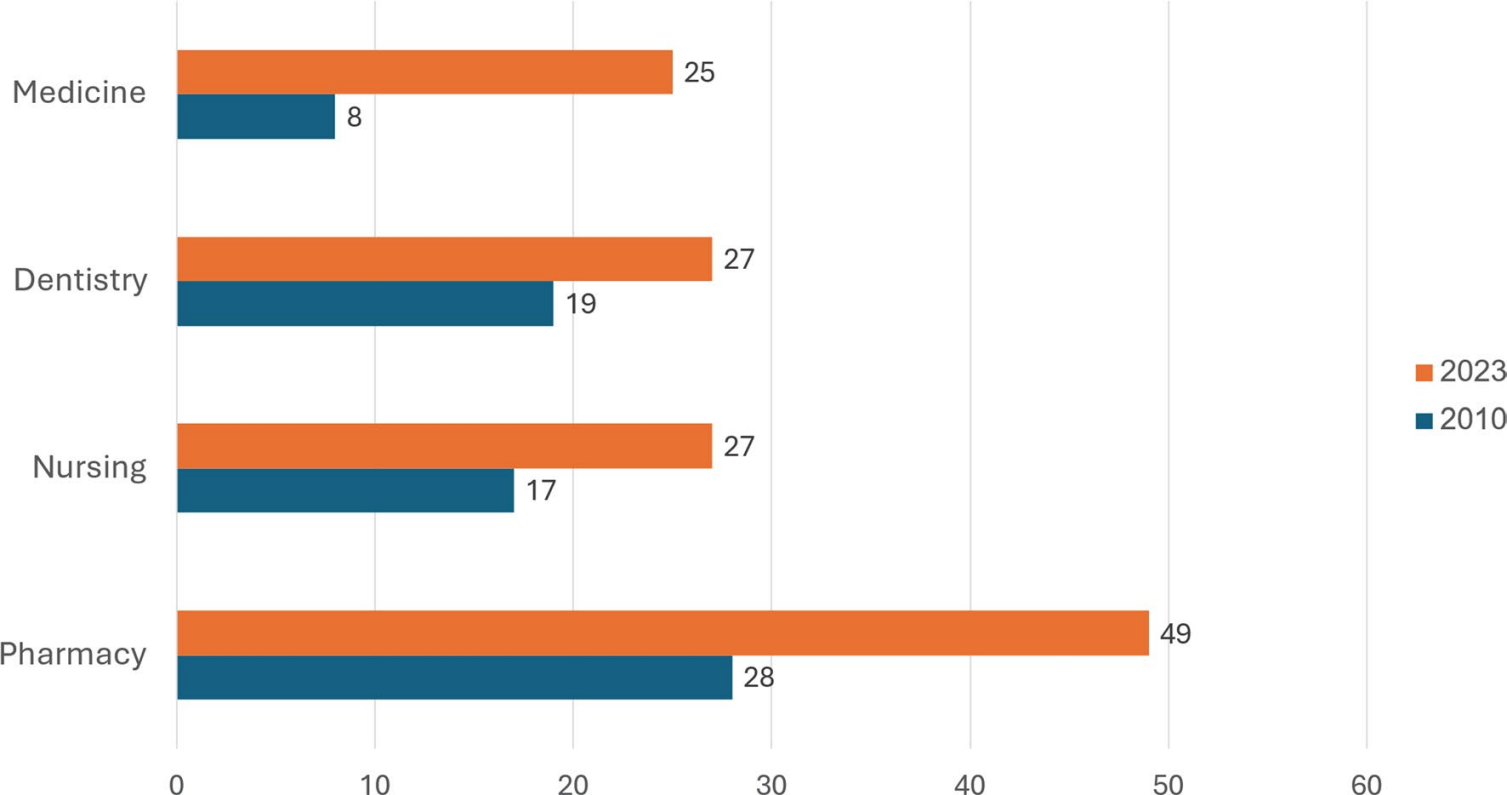
Percent of working professionals who are Arab, in selected health care professions, 2010 and 2023

In 2023, among employed Israelis up to age 67, Arabs constituted approximately one-quarter of the physicians (25%), nurses (27%), and dentists (27%), and half of the pharmacists (49%). These percentages are all much higher than they were in 2010, with the difference being particularly marked in the case of physicians (Fig. 2). Those percentages are also greater than the share of Israeli Arabs in 2023 employment in all the academic professions^9^ taken together (10%) and 2023 employment overall (16%),^10^.^11^

**Fig. 3:**
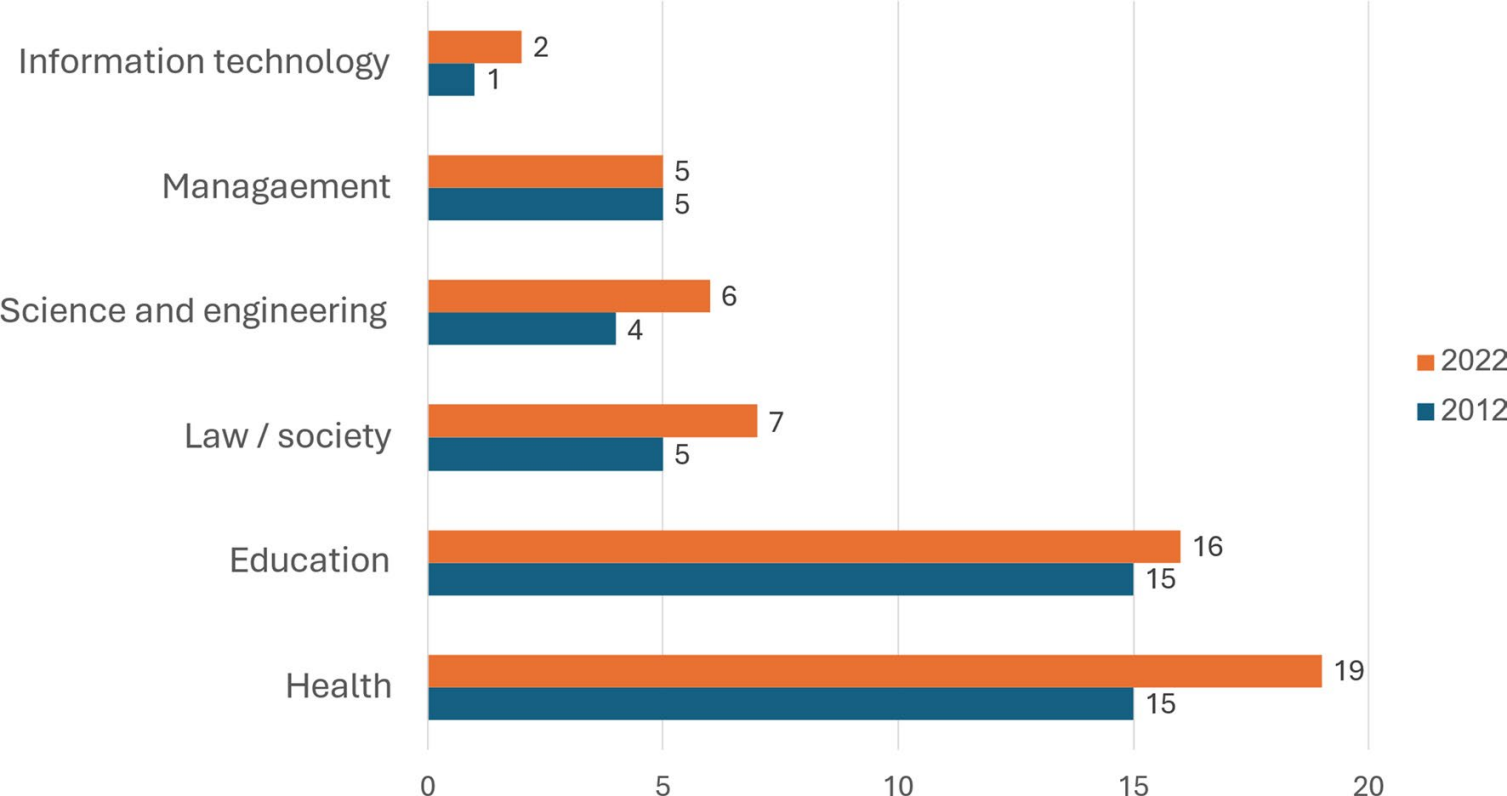
Percent Arabs in key professional groupings, 2012 and 2022

As indicated in Fig. 3, in 2022 Israeli Arabs constituted 19% of Israelis working in any of the health professions (i.e., not just the four professions which are the focus of this article). The only other grouping of professions to come close to this was education (16%), while Arab representation was much lower in the law/society, science and engineering, management, and information technology groupings. Already in 2012, education and health were the two groupings with the highest percentages of Arab professionals, but in 2012 the percentage was identical for those two groupings (16%). The four percentage point increase for health between 2012 and 2022 was the highest among all the professional groupings.

Supplementary Table 1 provides addition data on these and other occupational groupings.

### All Licensed Professionals Up to Age 67

**Fig. 4:**
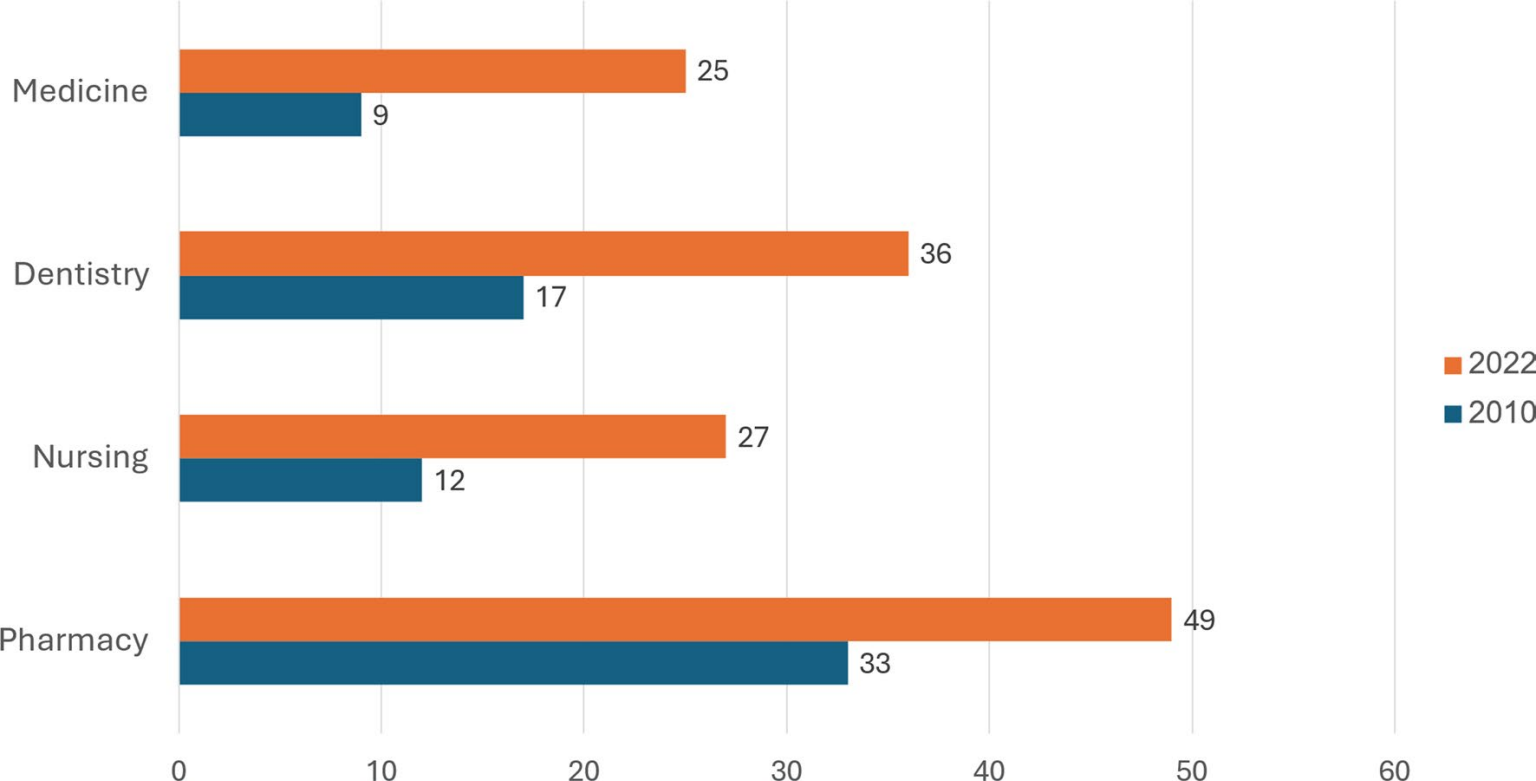
Percent of all licensed professionals up to age 67 who are Arab, in selected health care professions, 2010 and 2022

For physicians, nurses, and pharmacists, in 2022 the Arab share in the stock of licensed health care professionals up to age 67 (i.e., irrespective of when they received their licenses, whether or not they studied in Israel, and whether or not they are working) was similar to their share in employment in the relevant field. In the case of dentists, in 2022 the share of Arabs among licensed professionals up to age 67 was higher than their share among employed professionals (36% versus 27%). In all the professions the shares were substantially higher in 2022 than in 2010 (Fig. 4).^12^

Figure 8 in Appendix D shows graphically the percentage of licensed professionals for all four professions studied and for all the years between 2010 and 2022.

### Newly Licensed Professionals

**Fig. 5:**
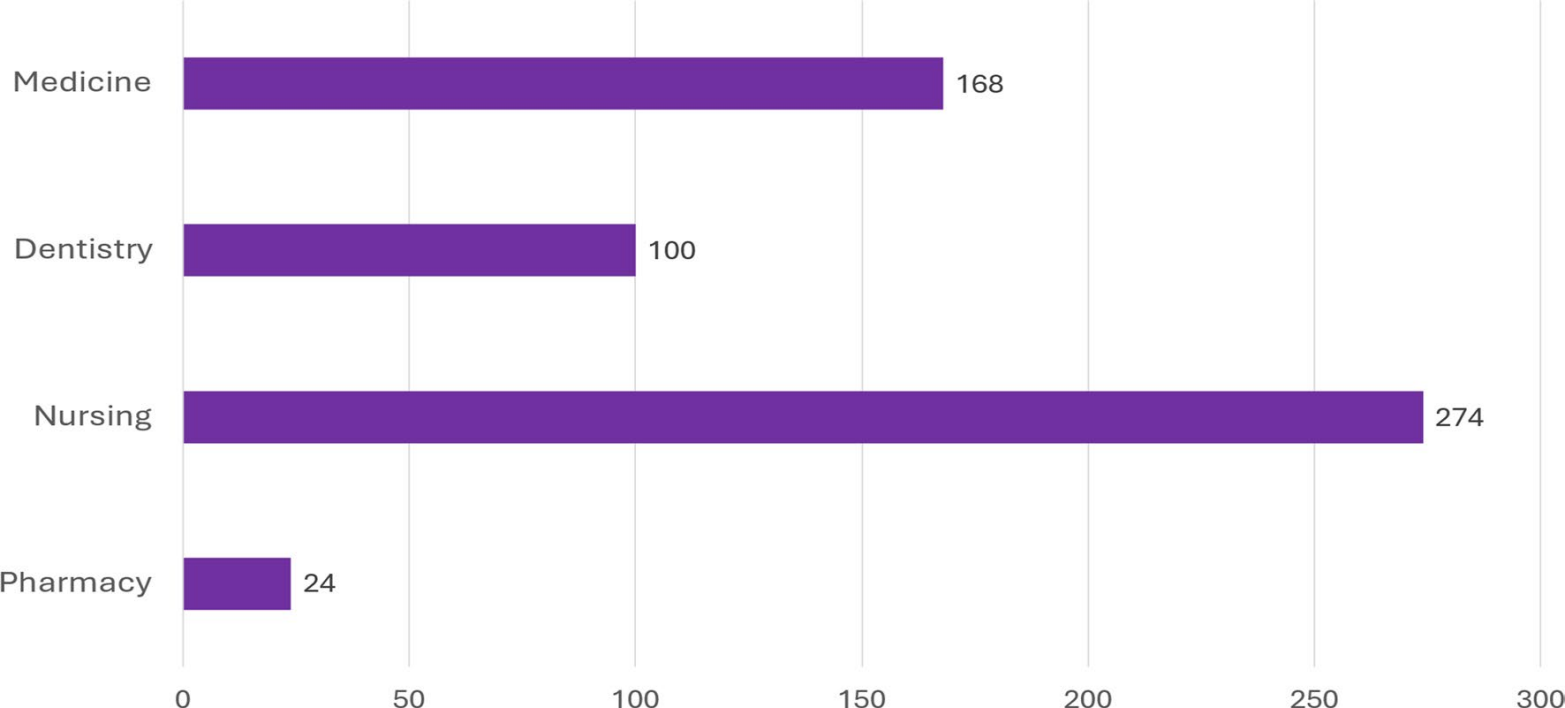
Cumulative percent growth in the number of newly licensed professionals, in selected health care professions, 2010–2022

Between 2010 and 2022, the number of new licenses granted annually to Israeli citizens (including both Arab and Jewish citizens), increased markedly in each of the health professions studied (Fig. 5). This was particularly true for medicine (in which the number of new licenses almost tripled over the 12-year period) and for nursing^13^ (in which the number of new licenses almost quadrupled). For both medicine and nursing, the number of newly licensed professionals grew fairly steadily over the twelve-year period, peaking in 2020 for nursing and in 2021 for medicine.

Supplementary Table 2 provides data on these growth percentages for Arabs and Jews separately. For example, it indicates that for nurses the percentage growth overall was 274%, while for Arabs it was 449%, and for Jews it was 199%.

**Fig. 6:**
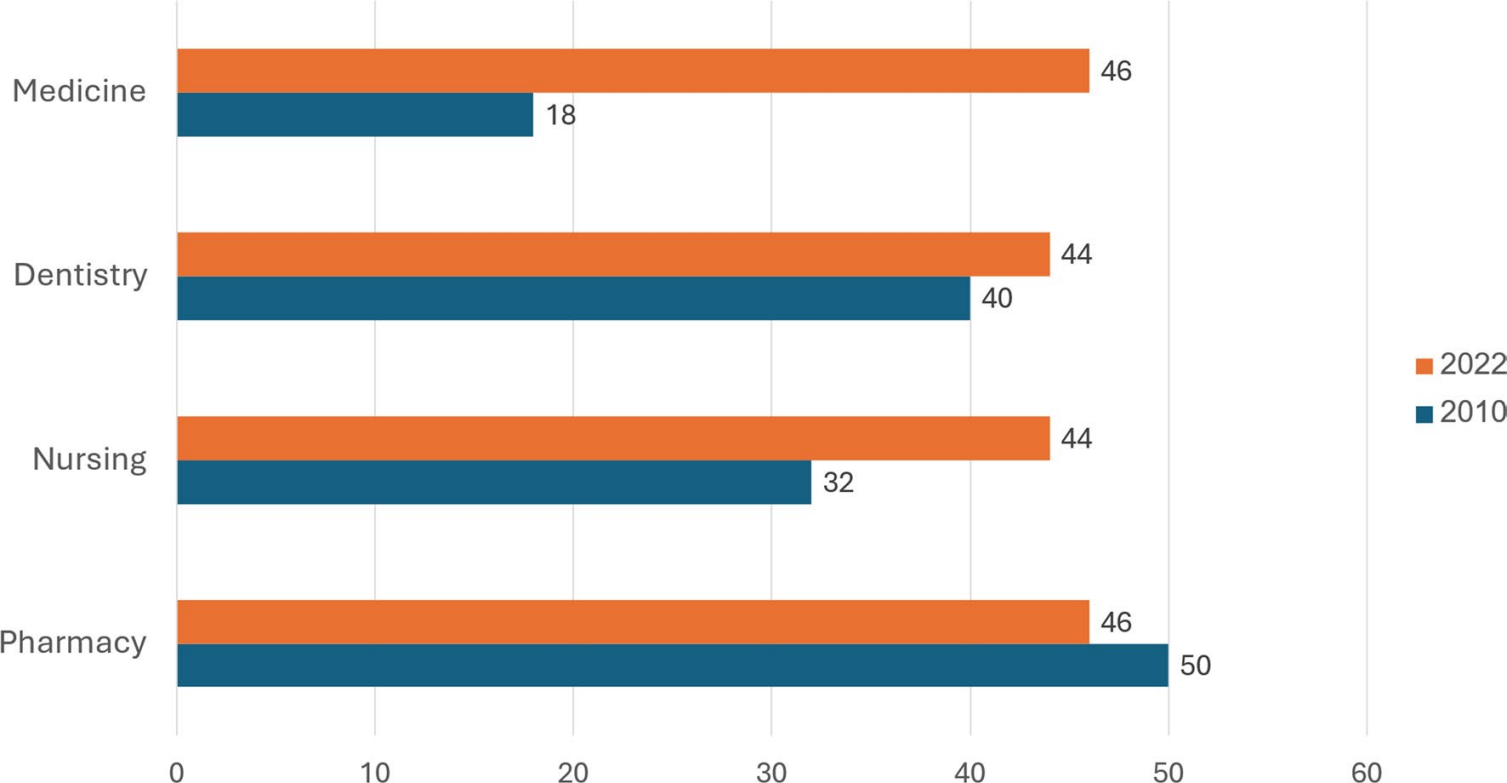
Percent of newly licensed professionals who are Arab, in selected health care professions, 2010 and 2022

Additional important findings regarding the past decade emerge when moving from the total for all citizens to an analysis by population group. As indicated in Fig. 6, the percentage of newly licensed professionals who are Arab has increased substantially among physicians and nurses and remained stable among dentists and pharmacists (Fig. 6).

To contextualize these findings about percentages, it is important to note that the absolute number of new licenses granted annually increased sharply for both Arabs and Jews in medicine, dentistry, and nursing, while in pharmacy there was a moderate increase in the number of new licenses among both groups (see Supplementary Table 2 at the end of this document).

### Professional Studies (in Israel and Abroad)

As indicated in Appendix E, professional studies in Israel and professional studies abroad are both important pathways toward subsequent licensure and employment in the health care professions. Indeed, in 2022 among licensed professionals up to age 67, the percentage that had studied in Israel was 42% for physicians, 50% for dentists, and 49% for pharmacists. Thus, in the case of those three professions between half and three-fifths had studied abroad.^14^

We consider the two pathways separately because they differ substantially regarding both the Arab–Jewish mix and the completeness of the available data.

### First Degree Students in Israeli Institutions of Higher Education

In the 2022–2023 academic year, Arabs constituted 17% of students in first degree programs at Israeli universities^15^—up from 15% in 2012–2013. At Israeli academic colleges (not including teacher colleges), Arabs constituted 24% of students in first degree programs in 2022–2023—up from 7% in 2012–2023.^16^

**Fig. 7:**
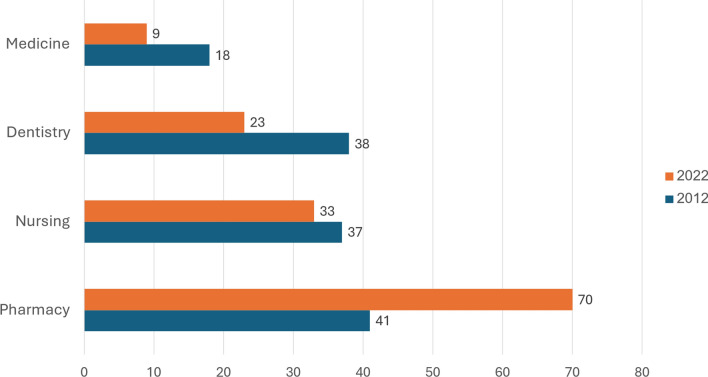
Percent of students who are Arab among all first-degree students in selected health-related departments in Israel

**Fig. 8:**
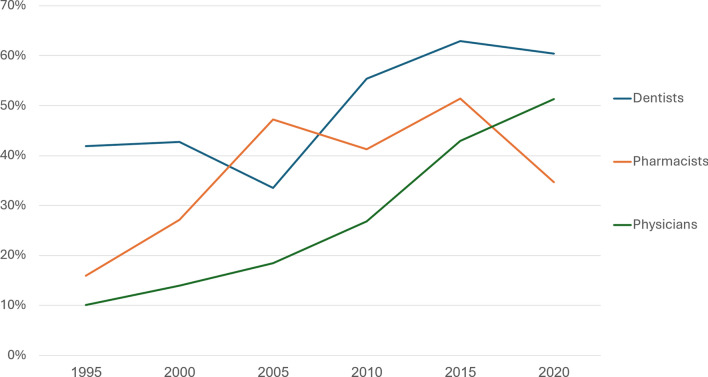
Place of training of newly licensed professionals, in selected health care professions, 2020–22 (Percents)

As can be seen in Fig. 7, in the 2022–2023 academic year, in Israeli institutions of higher education (including both universities and colleges), the percentage of first-degree students who were Arab was 70% in pharmacy, 33% in nursing, 23% in dentistry, and 9% in medicine. Between 2012/3 and 2022/3 the percentage of first-degree students in Israeli institutions who are Arab increased substantially for pharmacy, declined slightly for nursing, and declined substantially for medicine and dentistry.

Table 1 provides important context for understanding these changes, with a focus on the universities (i.e., not including colleges). It shows that, while the number of first-degree university students overall increased by 19% between 2012/3 and 2022/3, the increase for Arab students was twice as large—38%. The table also shows that the greatest percentage increases in the number of students were in three clusters (law and business, natural sciences, and architecture/engineering) and that the increases in these clusters were particularly pronounced among Arab students. In the health professions^17^ there were only modest increases, while the number of students declined substantially in the humanities, education, and the social sciences.

**Table 1:** The percentage change in the number of first-degree students at universities between 2012/3 and 2022/3, by departmental clusters. *Source*: Council for Higher Education

	All students (%)	Arab students
Total	19	38
Humanities	− 36	− 20
Education	− 33	0
Social sciences	− 6	− 42
Law and business	78	236
Health professions	20	7
Natural sciences	68	131
Engineering and architecture	44	245

As indicated in Supplementary Table 3, the high percentage increases for Arab students cannot be explained by low levels in 2012.

### Professional Studies Abroad

As indicated in a recent inter-ministerial report [15], in 2019 22% of Israeli Arabs studying in institutions of higher education did so outside of Israel. Moreover, most of them were studying in the health care field. According to that report, the main reason for studying abroad was difficulty in securing acceptance to an Israeli institution, which in turn was due to inadequacies in Hebrew language skills, grades in matriculation exams, and psychometric scores. The report also indicates that, across several health care professions, Israeli Arabs who studied outside Israel subsequently have difficulties passing the licensure exams and securing entry-level positions in the professions (including residencies). These difficulties are attributed to the low quality of some of the professional schools in other countries, insufficient clinical exposure during the degree programs, limited Hebrew language skills, and insufficient familiarity with the Israeli health care system.^18^

Comprehensive data are not available regarding the Arab–Jewish mix of licensed professionals who studied abroad. However, we do have various indications that study abroad is more prevalent among Arabs than among Jews.

For example, Arab physicians are more likely than their Jewish counterparts to have completed their professional studies outside of Israel. In 2022, among licensed physicians up to age 67, 68% of Arabs had studied abroad, which is higher than the overall rate (58%) and the rate among Jews (54%) [55]. Among physicians who received licenses in 2021, 48% were Israelis who had studied abroad [42], and it is estimated that 70% of them were Arabs [15].

We also know that among professionals who received their licenses in 2022, 8% of physicians, 17% of nurses, 24% of dentists, and 32% of pharmacists had studied at non-Israeli professional schools in the continents of Africa and Asia [42, 56]. These were predominantly in the Palestinian Authority, Jordan, and other Arabic-speaking countries.^19^ While we do not have data on the Arab–Jewish mix of students at these schools, a reasonable guestimate is that all (or almost all) of the students from Israel in Arabic-speaking countries were Arab. This would be in addition to Arab students studying in other continents, especially Europe.

## Discussion

Israeli Arabs currently constitute a large and recently increased share of the Israelis employees in four key health professions.^20^ Accordingly, Arab health care professionals play a major role in the provision of health care services in Israel, caring for both Arab and Jewish patients.

In this discussion section we consider: (1) Features of Israeli health care that have contributed to the relatively high percentage of Israeli Arabs currently employed in four key professions; (2) Factors contributing to the growth of employment of Israeli Arabs in key health professions over the past decade; (3) The importance of developing an integrated story across key parameters; (4) Similarities and differences across the key professions; (5) Professional studies outside of Israel; (6) Several remaining (and long-standing) challenges for Arabs in the health care professions; (7) A newly emerging challenge; (8) Directions for further research; and (9) How the role of Israeli Arab professionals could become even more significant if, and when, regional tensions abate.

1. *Features of Israeli health care contributing to the extensive employment of Arab in four key health care professions.*
  Our overview indicates that as of 2023, the share of Israeli Arabs in employment in the four health care professions reviewed—medicine, dentistry, pharmacy, and nursing—ranged from 25 to 50%. These shares are all greater than the share of Israeli Arabs in the employment in all the academic professions taken together (9%) [57].

Israeli health care has several features that contribute to the extensive employment of Arab health care professionals and the special role of those professions as a vehicle for social mobility for Israeli Arabs. Some of these features are characteristic of health care in general, while others are specific to Israeli health care.

One of these features is the humanistic and universal values of health care and the health care professions [14, 58], as reflected in the Hippocratic Oath taken by physicians, and Israel’s National Health Insurance Law. This may well contribute to the interest of Israeli Arabs in working in the health care field, the interest of Israeli hospitals and health plans in employing Israeli Arabs, and the generally harmonious interactions among patients and professionals of different nationalities and ethnicities. The humanistic and universal values of health care probably also contribute to health care status as a setting for strong cooperation among Jewish and Arab professionals,^21^ and a sphere in which the provision of services largely transcends differences in religion or nationality between service provider and service recipient [59]. These values also contribute to the emphasis placed on merit in promotion decisions and the higher level of trust in the health system among Israeli Arabs than among Israeli Jews [60, 61].

A second key feature is that Israel operates a single, integrated national health insurance system which covers all its citizens [14], which is based largely on public financing, and which includes a universal right to accessible and affordable health care [62]. As financing is predominantly public rather than private, and as health care is a right, hospitals and community-based health care providers have both a responsibility, and are supposed to get the funds needed, to employ a full complement of health care professionals in Arab localities (even though residents of Arab localities tend to have below-average disposable incomes). Efforts underway to improve the provision of health care in Arab localities [50] are likely to increase the need to encourage more Israeli Arabs to work as healthcare professionals.

A third relevant feature is the importance of cultural understanding, connection, and competence in the delivery of health care [63]. While Arab and Jewish clinicians can—and very often do—treat both Arab and Jewish patients, Arab clinicians are usually best prepared to provide culturally competent care of Arab patients. Hospitals and community-based health care providers understand this, and it influences their hiring practices, particularly in community care clinics situated in Arab localities.

A fourth relevant feature is that, in light of the above, the health care professions offer employment opportunities both in settings within Arab cities and villages and in mixed, or even predominantly Jewish, cities (including those in the peripheral regions, where most Israeli Arabs live). Thus, these professions can appeal both to Arabs who wish to work within the Arab community and to those who wish to work in multi-cultural settings (and to those who want to preserve both options).

A fifth relevant feature is that Israel does not train enough health professionals to meet its needs (particularly in medicine and dentistry) and accordingly Israeli relies heavily on professionals trained abroad.^22^ This has created opportunities for Israeli Arabs, who have demonstrated substantial willingness and ability to train outside of Israel—in Europe, in nearby Arab countries, and in the Palestinian Authority.^23^ This pathway has allowed many Israeli Arabs to circumvent barriers to entry in Israeli institutions; however, it is also associated with difficulties in passing the licensure exams and in securing employment [15].

A sixth relevant feature is that healthcare professions carry high prestige in Arab society and are viewed as pathways to social mobility [41]. These careers allow professionals to serve their communities while achieving personal financial stability and contributing to local economic development. Moreover, as healthcare professions allow Arab professionals to work within Arab localities, they can reduce commuting times and reduce exposure to potential discrimination in the broader Jewish-dominated economy. For all these reasons there is strong family and community encouragement for the pursuit of healthcare careers [20].

Additional relevant features include encouragement and support from family members [40]; opportunities to help the Arab community [64]; the difficulty Arabs face in securing employment in several lucrative industries outside of health care (such as high-tech and defense) [14] as well as the large law and accounting firms [65]; the greater willingness of Arab males than Jewish males to work as nurses; the particularly high status of medicine in the Arab population [66]; and the tendency of Jewish pharmacy graduates to prefer employment in a pharma company rather than in health care.

Thus, health care has quite a few features that have made it more accessible to Arab professionals than some other areas of social and economic activity. We believe that it can, nonetheless, be a source of inspiration, encouragement, and perhaps even practical lessons, beyond health care. An example of one such lesson is that Arab professionals can deliver high-quality, well-appreciated, care to both Jewish and Arab patients in addition to their unique capacity to provide culturally responsive care to Arab patients. Hopefully, a broad range of professions and industries will be able to build on the progress that has been made in integrating Arab professionals in the Israeli health care workforce.

2. *Factors contributing to the growth of employment of Israeli Arabs in key health professions over the past decade.*
  This overview has shown that the share of Israeli Arabs among employed health professionals has increased over the past decade for all four of the health professions covered. Here, too, many of the contributory factors have also been common to most, if not all, of the four professions. As detailed below, they have apparently included systemic developments, developments specific to the Jewish population, and developments specific to the Arab population. Moreover, some of the developments primarily affected demand for health professionals, while others primarily affected the supply of health professionals, as detailed below.

**The key development at the systemic level** was a growing shortage of health professionals. This was due primarily to an increase in need and **demand**, as a result of population growth and population ageing. In addition, the expansion of national health insurance to include dental care for children and the elderly, also contributed to the growth in demand for dental care and hence for dentists. There was also a growing government recognition of the need to increase the number of physicians and other health professionals in the periphery, which is where most Israeli Arabs live [64, 65].

Other system-level developments (or lack of developments) took place on the **supply** side of the market for health care professionals. Israeli institutions of higher learning substantially increased the number, size, and diversity of nursing schools in Israel (including schools in the periphery and schools in which Arab was the language of instruction). In contrast, during the 2010–2022 period, training programs for physicians and dentists in Israeli institutions of higher education did not expand in parallel with the increase in demand for those types of health professionals.

Another supply-related development was that the Israel Medical Association secured a major increase in physician wage levels in its 2011 collective bargaining agreement with the major employers of physicians.

**The key development specific to the Jewish population** during the 2010–2022 period was the retirement of many Jewish professionals who had immigrated to Israel from the Former Soviet Union around 1990—a development with clear implications for overall **supply**. An additional supply side development was the growth of Israel's high tech sector, which offered salaries well above those offered in the health care sector. While it has been difficult for Israeli Arabs to break into the high tech sector, that sector has drawn in many highly talented young Israeli Jews who might otherwise have opted for careers in the health care sector.

**Several developments specific to Israel’s Arab population** also played a major role in facilitating the growth of employment of Israeli Arabs in the health professions, as follows:

A series of multi-year government initiatives to promote economic development in the Arab sector allocated resources to improve access to higher education and professional training for Israeli Arabs [22]. These funds were used to finance preparatory programs as well as scholarships, and targeted support for underrepresented groups. As noted above, the number of Israeli Arabs in Israeli higher education doubled between 2009 and 2022 [48], with significant growth in healthcare-related fields—thus enhancing the **supply** of Arab health care professionals.

The number of Israeli Arabs studying health professions abroad also grew significantly. This probably reflected a growing capacity of Arab families and extended families to finance such studies and a recognition of the growing employment opportunities for Israeli Arabs within health care (in contrast to high tech which remained difficult to break into). The major increase in physician wage levels probably also boosted the willingness of young Israeli Arabs and their families to invest in studies abroad.

Sociological developments within Arab society probably also played a role. These have led to an increase in the number of Arab women in the health professions, particularly in dentistry-another supply side factor (See Supplementary Table 4).

As such the developments within health care in the 2010–2022 period constitute a continuation of an earlier and broader trend which has been documented by Haider and Bar-Haim [24]. They found that a key element in the gradual improvement in the socioeconomic status of Israel’s Arab minority has been its ability to resourcefully take advantage of opportunities—including unmet national needs.

Irrespective of what has caused the changes, the increased representation of Israeli Arabs across all four examined health professions over the past decade is a noteworthy finding. This trend aligns with broader societal shifts towards greater integration and social mobility for the Arab minority in Israel (despite several recent policy setbacks and significant remaining challenges in how the State of Israel relates to its Arab citizens). The particularly marked increase in medicine and nursing suggests that these fields may be serving as important pathways for social and economic advancement.

3. *The importance of developing an integrated story across key parameters.*
  Comparisons of the percent Arab across key parameters—employment, licensure, and professional education—underscore both the value of considering these parameters in an integrated manner and the importance of bearing in mind even those parameters for which data are incomplete (such as the extent of professional education abroad):
  The substantial increase over the past decade in the Arab share in the stock of licensed professionals is too great to be accounted for solely by the increase in their share of the inflow of newly licensed professionals. Apparently, the predominance of Jewish professionals in the outflow (retirement, etc.) is also an important part of the story.
  Arab professionals account for similar shares in the number of employed professionals and the number of licensed professionals. This suggests that, historically, the ability of licensed professionals to find employment has been similar—and very high—for both Arabs and Jews.^24^ (An important exception is that in recent years it has taken newly licensed Arab physicians longer than their Jewish peers to find residency positions, particularly if they studied medicine outside of Israel [67].)
  In general, Arabs account for a larger share of newly licensed professionals than they do of first-degree students in the relevant departments in Israeli universities. This underscores the Arab population's heavy reliance on studies outside of Isael.
  A related point is that over the past decade the share of Arabs among newly licensed professionals increased substantially for physicians despite the concomitant decrease in their share among first-degree students in medicine at Israeli institutions of higher learning. The discrepancy is apparently due to a growth in the role of studies outside of Israel.^25^

Supplementary Table 5 brings together in one place the key findings of this paper regarding all the key parameters and all the health professions covered in this paper. Appendix F illustrates how stocks and flows are related, using physicians as an illustrative example.

4. *Similarities and differences across professions.*
  This study contributes to the existing literature by providing a comprehensive, multi-professional perspective on Israeli Arab integration in health care. While previous studies have focused on individual professions or specific aspects of the issue, our research offers a comparative view across four key health professions, allowing for a more nuanced understanding of trends and patterns.

As noted above, many of the features of the Israeli health care system that contribute to the relatively high current percentages of Israeli Arabs in the health care professions and to the increases in those percentages of over the past decade, apply to all four of the professions covered: medicine, dentistry, nursing, and pharmacy.

At the same time, there are important differences across these professions. Some of these differences have been noted above, such as the centrality of study abroad. Other important differences include the cutoff levels for admission to degree programs in Israel, the extent to which qualified Jewish young people are seeking to enter those degree programs, and the extent to which those degree programs are fully subscribed. As demonstrated in this article, a multi-professional overview such as ours can inform, but also needs to be supplemented by, more in-depth examinations of particular professions. These should include in-depth interviews to get at the reasons behind key phenomena, such as study abroad. Examples of profession-specific findings of this study which call out for more in-depth consideration include:

- **Pharmacy** is the health care profession in which Arabs constitute the largest percentage
- **Medicine** is the health care profession in which the percentage of Arabs has grown the most
- **Dentistry** is the health care profession which is the most reliant on professional studies outside of Israel
- **Nursing** is the health care profession which is the fastest growing as well as the one that is least reliant on professional studies outside of Israel^26^

Note that only some of the unique characteristics noted in the list speak directly to the Arab–Jewish mix: those regarding pharmacy and medicine do so, while those regarding dentistry and nursing do not. The latter are nonetheless highly relevant to the Arab–Jewish mix, through indirect pathways that need to be explored further.

5. *Professional studies outside of Israel.*
  Study outside of Israel, especially in neighboring jurisdictions and Eastern Europe, has been a very important means by which many Israeli Arabs have been able to qualify as healthcare professionals. The reliance on studies outside of Israel is set to continue in the years ahead.

Study outside of Israel highlights the complex interplay between domestic educational capacity, opportunities abroad, and professional aspirations. Many of the Israeli Arabs who study outside of Israel (particularly in the cases of medicine and dentistry) due so primarily because they are unable to meet the very demanding entry requirements of the professional schools within Israel. Studies in recognized professional schools outside of Israel suffice for licensure and employment. However, graduates of many of those schools find it difficult to secure employment in Israeli health care, particularly in the more prestigious institutions and specialties. Moreover, few of the graduates of non-Israeli professional schools progress to leadership positions.^27^ Accordingly, it will be important to provide greater opportunities for Israeli Arabs to prepare for careers in the health professions via studies in Israeli institutions of higher education. Institutions in the peripheral regions (where most Israeli Arabs live) are particularly well situated for this.

6. *Continued long-standing challenges for Arab professionals within health care.*
  Even within Israeli health care, many challenges remain. Arab health care professionals are under-represented in senior and managerial positions [14] as well as in the most prestigious medical specialties (ref).^28^ In addition, the achievements in the health professions that have characterized Arab Israelis living in the north and center of the country are less prevalent among the Bedouin population living in the south (ref).^29^ Moreover, in times of increased conflict, health care institutions are not immune to the general increase in Arab–Jewish tensions, and this can impact both interactions among professionals and interactions between professionals and patients [27, 33, 35, 41, 43, 69, 70]. In particular, the Israel–Hamas War has been a major source of tensions on hospital wards between Arab and Jewish professionals, and a major Ministry of Health initiative had been launched to address those tensions [71]. Another key challenge is that Arabs are significantly under-represented in the mental health professions (particularly psychiatry and psychology, though less so in clinical social work) [72]. This is problematic, as cultural and linguistic diversity are even more important in these professions than they are for the health professions in general [72–75]. There are also substantial shortages of Arab professionals in the fields of communication/speech therapy, physical therapy, and occupational therapy [76]. As a result, Arab patients in need of these services (and particularly those who live in Arab villages in the Galilee or the Negev) tend to have impaired access to these important services.^30^
7. *A newly emerging challenge.*
  There is a major new challenge on the horizon with respect to study abroad. Starting in 2026, Israelis who studied abroad will only be able to take the medical licensure exam if they have studied at a medical school deemed to meet various quality standards [51].^31^ Further information about the background and objectives of this change, known as the Yatziv reform, can be found in Appendix G.^32^

The Yatziv reform will have a large and disproportionate effect on Israeli Arabs. Among physicians who received their licenses between 2020 and 2022 (excluding physicians who pursued medical studies in conjunction with the Israel Defense Forces (IDF) and immigrants to Israel), 76% of the Jews and 10% of the Arabs studied medicine in Israel. In contrast, the percentages who studied in a non-Israeli medical school recognized by the Yatziv reform were 20% for Jews and 30% for Arabs. Most significantly, the percentages of new licensees who studied in a non-Israeli medical school not recognized by the Yatziv reform were 5% for Jews and 60% for Arabs [67].^33^

It is important to note that the extent to which Arabs have studied medicine abroad (and particularly at medical schools not recognized by the Yatziv reform) grew significantly over the past decade. As a result, while among all Arab licensees up to age 67 in 2022 68% had studied abroad, among those who received their licenses between 2020 and 2022 84% had studied abroad,

While the Yatziv reform will have only a gradual effect on the stock of all physicians up to age 67, it will sharply reduce the number of newly licensed physicians—the group of physicians who are critical for filling internship and residency positions [15]. The shortfall is expected to impact peripheral hospitals particularly hard. To address those challenges, several initiatives are already underway to increase the number of Israeli medical schools and increase the number of slots for students in existing Israeli medical schools. Other initiatives are underway to increase the ability of Israelis living in the periphery (which includes a large concentration of Arabs) to secure entry into Israeli medical schools and successfully complete their medical studies there [15, 51]. In addition, there is an initiative to provide substantial financial support to Israelis who study abroad in medical schools recognized by the Yatziv reform [15].

No less importantly, the government has launched an initiative to upgrade the skills of recently licensed physicians who had studied at lesser quality medical schools [15, 51].

Study abroad has been critical for Arabs pursuing careers in dentistry, with many studying at dental schools considered to be of high-quality [15]. Looking ahead, it will be important to preserve the high-quality options for study abroad and also to provide greater opportunities for Israeli Arabs to prepare for careers in dentistry via studies in Israeli institutions of higher learning.

8. *Directions for further research.*
  Directions for further research, in light of this study’s findings, include:
  How will the percentage of Arab Israelis among those receiving medical licenses be affected by the Yatziv reform, as well as the various initiatives underway to increase study opportunities at Israeli medicals schools and recognized foreign medical schools?
  To what extent are growing opportunities in high tech and other STEM-related fields drawing away particularly talented Jewish and Arab young people from the health care professions?
  For each of the four fields covered in this paper—medicine, dentistry, nursing, and pharmacy—what are the inter-relationships between employment, licensure, professional studies, and other key parameters not covered in this paper?
  Why is it that pharmacy is characterized by a substantially higher representation of Arabs than the other major health care professions?
  To what extent are there male–female differences, and differences between cohorts, in the key trends noted in this paper regarding Arab employment, licensure, and training in the health professions?
  To what extent are the Bedouin, Druze, and East Jerusalem populations taking part in the successes in the health profession that have characterized the overall Israeli Arab population?
  Within each of the major health care professions, to what extent are there Arab–Jewish differences in incomes, places of employment, recognition as specialists, and advancement into leadership roles?
  Why are there shortages of Arab health professionals in such fields as psychology and speech therapy, and what can be done to address them?
  To what extent have Israeli Arabs progressed in non-health professions such as law and engineering, and what might other sectors learn from the health sector?
  To what extent have the healthcare professions, relative to other professions, contributed to the increase in the proportion of Arabs in the middle class since 2000?
  How could qualitative methods be used to further explore the lived experiences of Israeli Arab health professionals and the motivations behind their educational and career choices?
  What can Israel learn from studies of the ethnic composition of the health care professions in other multi-ethnic countries?
9. *Looking to the future.*
  While much remains to be done, achievements to date in the role of Israeli Arabs in the health care professions remind us that even in these challenging times of war and internal tensions within Israel there are substantial areas of Arab–Jewish cooperation and co-existence. Hopefully, in the years ahead the current conflicts and tensions will abate, and we can all more fully realize the benefits of a shared Israeli health care system and a shared Israeli society.

Moreover, over the years, there have been ebbs and flows in the number of patients from the Palestinian Authority and Arab countries arriving in Israel for care in Israeli tertiary hospitals [78]. The same is true regarding the number of health care professionals from the region coming to Israel for advanced training [79]. Hopefully, if, and when, tensions in the region decline, more Arabs from outside of Israel will be able to take advantage of treatment and training opportunities in Israel. In that scenario, Israeli Arab health care professionals would be able to play a particularly important role in meeting the increased demand for such treatment and training, due to their Arabic language skills and their familiarity with Arab cultural norms.

## Policy Implications

The substantial and increasing representation of Israeli Arabs in four major healthcare professions constitutes an important achievement, but challenges remain. To build on achievements to date, and to promote continued progress, a multi-pronged approach is needed. This should include expanding access to health professional education within Israel, upgrading the skills of graduates of non-Israeli universities, promoting diversity in leadership positions and key specialties, and expanding specialty care services in Arab localities. A comprehensive policy effort of this sort would not only benefit the Israeli healthcare system but could also serve as a model for other countries facing similar challenges in professional integration and minority representation.

More specifically, we encourage policymakers to:

1. **Expand domestic educational access:** Many Arab high school students who have the potential to become effective health care professionals have difficulties gaining acceptance into Israeli professional schools. Recent national initiatives to expand the total number of study slots in Israeli medical and nursing schools, along with recent initiatives to bring in more students from peripheral regions, will be helpful in this regard [55]. These should be supplemented by initiatives that focus on Arab students. Such policy initiatives should include increasing the number of scholarships for Arabs students in university departments related to the health care professions,^34^ expanding preparatory programs for Arabic-speaking students, and revising admission criteria to be more inclusive.
2. **Maintain and expand efforts to support skills development for graduates of foreign institutions:** Many Arab health professionals have obtained degrees abroad, and some of them face challenges with licensure and/or employment. To address this, the Ministry of Health has recently introduced a comprehensive upgrade program for those who studied medicine abroad in lesser-quality institutions, whether those recent graduates are Jews or Arabs [15, 55]. The upgrade program focuses on bridging clinical gaps, improving language proficiency, and preparing professionals for licensure exams and job placement in Israeli health care institutions. Serious consideration should be given to launching parallel programs for additional health professions.
3. **Promote diversity in leadership roles:** Despite the increase in overall representation, Arab professionals apparently remain underrepresented in leadership roles. The recent expansion in the number of Arab professionals has expanded the pool of relatively young potential leaders. Policies and programs aimed at mentorship, leadership training, and fair promotion practices should be developed.
4. **Enhance the availability of specialty medical care in Arab localities:** Many primary care clinics are already situated in Arab localities, but this is not the case regarding specialty care clinics. This has made it more difficulty for Israeli Arabs to access specialty care, even for the more common specialties. The recent growth in the number of Arab physicians—many of whom are pursuing specialty training—creates an opportunity to address this problem. The health plans can play a key role in this by establishing more ambulatory specialty care centers in the relevant districts and localities. In addition to improving access to health care, this would provide a boost to the local economies.
5. **Identify and address under-representation in some of the specialties**: Greater attention needs to be given to the representation of Israeli Arabs in specialty training programs in dentistry and some medical specialties, and in advanced practice training programs in nursing. Attention should also be given to other health professions in which Arabs are underrepresented, such as psychology and speech therapy. Possible interventions include recruitment initiatives and training incentives.
6. **Broaden global relevance:** The findings from this study have important lessons for other countries struggling with minority representation in healthcare. Policymakers should explore collaborations with international institutions, share best practices, and adopt a culturally competent approach to workforce diversity. While these insights are drawn from the Israeli context, they offer valuable guidance for promoting diversity and inclusion in healthcare globally.

## Conclusions

This study provides policymakers, educators, and healthcare administrators with a comprehensive overview of trends in Arab representation across multiple health professions, facilitating informed decision-making and policy formulation. By highlighting the role of education abroad in the professional development of Israeli Arab healthcare workers, this study can inform discussions on domestic educational capacity. Finally, this study contributes to the broader global discourse on minority representation in critical professions and the impact of workforce diversity on service delivery.

As societies worldwide grapple with issues of diversity, equity, and inclusion, the insights gained from this study may offer valuable lessons for other multicultural contexts facing similar challenges in professional integration and representation. At the same time, simplistic extrapolations from Israel to all other countries should be avoided due to several features of the Israeli situation which are not universal, such as its geographic location adjacent to countries in which the dominant language is the same as that of its main minority population.

As illustrated by this overview, studies of the representation of Israeli Arabs (and other minority groups) in the health care professions can benefit from an integrated approach that considers jointly several professions and which looks across the various stages in the professional pipeline, from professional studies through licensure and on to employment. This sort of integrated approach can also contribute to policy development regarding the health care professions.

The key finding of our study is that Israeli Arabs play a substantial and growing role in four major health care professions. We believe that this success has improved the availability and cultural responsiveness of Israeli health care and that it has also fostered stronger relationships between Arabs and Jews (both among employees and between employees and patients). The achievements in the health care professions have also contributed significantly to the growth of the Arab middle class and the broader Arab economy. Accordingly, it is crucial to develop policies that continue supporting Arab representation in healthcare, as this will benefit not only the Arab community but also Israeli society, societal cohesion in Israel, and the Israeli economy overall.

While this study focuses on the Israeli context, its findings have relevance for other multicultural societies grappling with issues of minority representation in professional fields. The success in increasing Arab representation in health professions, particularly in medicine and nursing, could offer valuable lessons for promoting diversity in other contexts.

## Supplementary Information

Additional file 1

## Figures and Tables

**Fig. 9:**
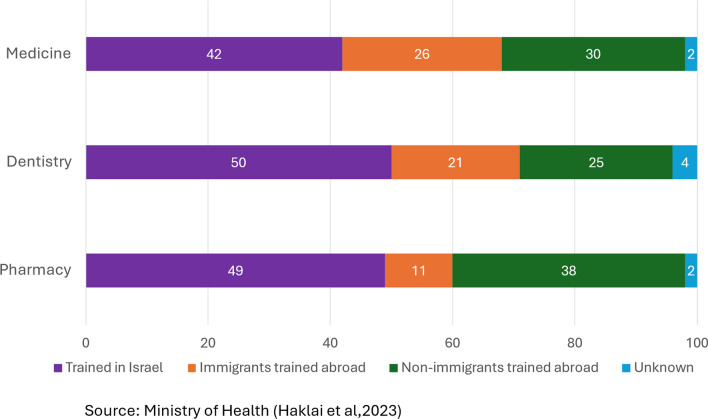
The percentage of licensed professionals for all four professions studied and for all the years between 2010 and 2022

**Fig. 10:**
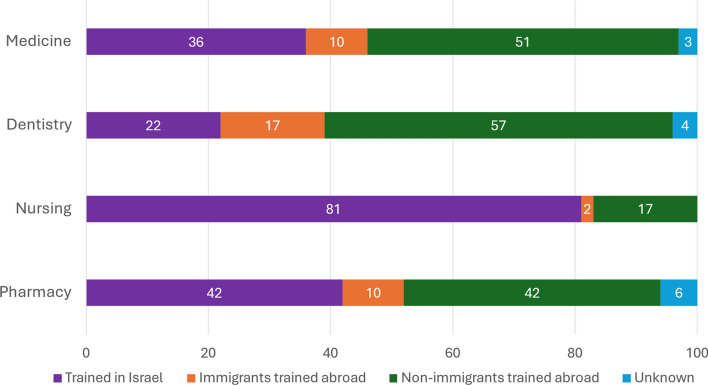
Place of training of licensed professionals up to age 67, in selected health care professions, 2022 (Percents)

**Fig. 11:**
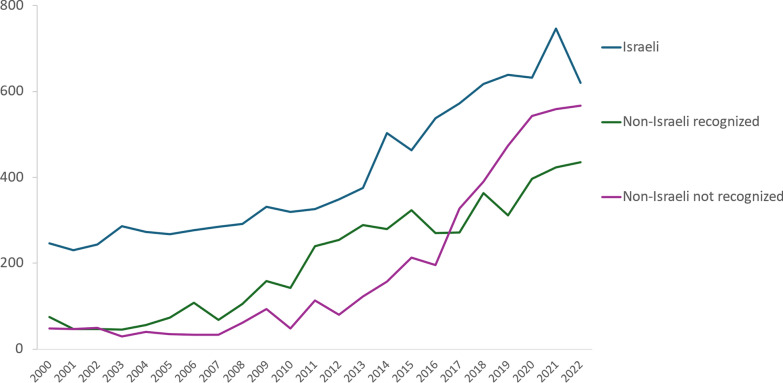
Percent of new licensees who were non-immigrants who studied abroad, for selected years

**Fig. 12:**
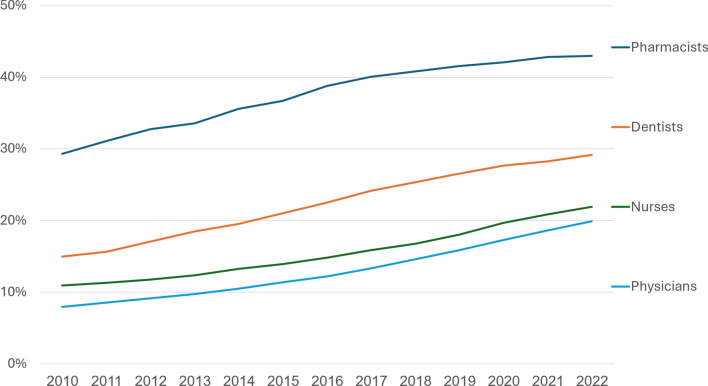
Medical licenses granted by year and by type of medical school

## Data Availability

Not applicable.

## Appendix A: The importance of workforce diversity in healthcare

As detailed below, workforce diversity in healthcare is important because it contributes to improved patient outcomes, addresses health disparities, promotes social cohesion, and creates economic benefits.

(A) **Improving patient outcomes.**
  Workforce diversity improves healthcare delivery by fostering culturally competent care that meets the needs of diverse patient populations. Arab healthcare professionals bring critical linguistic and cultural skills, enhancing communication and trust with Arab patients [63]. Studies show that patients are more likely to follow treatment plans when they perceive their providers as understanding their cultural background [34]. In Israel, Arab healthcare professionals play a dual role by providing high-quality care to Arab patients in their native language; and serving Jewish patients with the same standard of professionalism, contributing to a more inclusive healthcare system.
(B) **Addressing health disparities.**
  Diverse healthcare workforces are associated with reduced disparities in access to care. Arab professionals often work in underserved areas, where they mitigate the effects of geographic and socioeconomic barriers to healthcare access. This directly improves health equity in Arab-majority localities and peripheral regions [35].
(C) **Promoting social cohesion.**
  The integration of Arab healthcare professionals fosters collaboration between Arab and Jewish colleagues, creating spaces of coexistence in a divided society. Hospitals and clinics, where professionals work toward shared goals, exemplify how diversity can enhance both professional and societal cohesion [41].
(D) **Creating economic benefits.**
  Workforce diversity contributes to economic development by integrating marginalized groups into high-value professions. In Israel, the increased participation of Arabs in healthcare has boosted the Arab middle class and strengthened the broader economy [20].

By addressing these areas, Israel can serve as a model for other multicultural societies grappling with the challenges and opportunities of professional integration.

## Appendix B: Israeli Arabs and psychology

**Relevant characteristics of the field of psychology**

1. In 2022, the number of working age licensed psychologists was 13,780 (Table 2).
2. Psychology is one of approximately twenty professions regulated by the Ministry of Health (MOH). The MOH licenses psychologists in six categories: clinical, developmental, rehabilitation, education, health, and occupational-organizational-social. Most psychologists are in the clinical category.
3. Licensure in psychology requires a second degree in that field plus a supervised internship and a licensure exam in Hebrew that includes an oral examination as well as review of treatment summaries.
4. In 2022, there were 1,222 first-year students in first degree programs, and 624 new licenses were granted in psychology. Data were not readily available regarding the number of students in second degree programs.
5. In recent years over 80% of new licensees studied in Israel.
6. Clinical, developmental and health psychologists tend to work in the health care system. Rehabilitation and occupational psychologists tend to work in the rehabilitation system and/or the health system. Educational psychologists tend to work in the educational system.
7. In Israel’s official classification of occupations, psychology is not included in the health field but rather in the social and legal field.
8. Psychotherapy is provided not only by psychologists but also by social workers with training in psychotherapy, and to a lesser extent by psychiatrists as well.
9. In the Council of Higher Education’s clustering of first degrees, psychology is included in the social sciences rather than in the health care field

**Table 2:** Percent Arabs for the key parameters featured in this article for psychology

	2010	2023
Employment	4%	8%
	2010	2022
Licensees to age 67	5%	7%
New licensees	7%	7%
	2012	2022
First degree students	7%	7%

## Appendix C: The theories in which the article is grounded

This study is grounded in three interrelated theoretical frameworks that collectively explain the representation of Israeli Arabs in the healthcare professions: **social mobility theory**, **cultural competence theory**, and (to a lesser extent)** intersectionality theory**. Each provides a lens to understand the dynamics of Arab participation, challenges, and opportunities in healthcare professions.

### Social mobility theory

Social Mobility Theory explores how individuals move within a societal hierarchy through opportunities in education and employment [52]. For Israeli Arabs, healthcare professions have become a significant path to upward mobility, offering stable, well-paid, and prestigious careers that have helped expand the Arab middle class [24]. Government initiatives, particularly 5-year economic development plans, have supported this mobility by improving access to higher education and professional opportunities [22].

### Cultural competence theory

Cultural competence theory emphasizes the importance of understanding and integrating cultural diversity within service delivery systems, particularly in healthcare [53]. It argues that a workforce reflective of the patient population enhances culturally sensitive care, improving health outcomes and patient satisfaction [63]. In Israel, the representation of Arab healthcare professionals is crucial for delivering effective care to the Arab population, as these professionals bring linguistic skills and cultural insights that enhance communication and trust between patients and providers [40].

Moreover, the universal and humanistic values of the healthcare professions, including their focus on meritocracy and patient care, have facilitated relatively harmonious Arab–Jewish collaboration in healthcare settings. These values align with the principles of cultural competence and underscore the unique role of healthcare as a model of coexistence in a divided society [41, 45].

### Intersectionality theory

Intersectionality Theory examines how multiple social identities—such as ethnicity, gender, and class—interact to shape individual experiences [54]. For Israeli Arabs in healthcare, ethnicity combines with factors like gender and geographic location to create unique challenges and opportunities. For instance, Arab women have increasingly entered nursing and pharmacy despite facing potential barriers as both women and minorities [45]. This framework also helps explain why Arabs remain underrepresented in leadership positions and certain specialties, highlighting persistent systemic barriers such as limited access to quality education.

These three frameworks together provide a comprehensive view of Arab participation in Israeli healthcare. Social mobility theory explains broader socioeconomic advancement, cultural competence theory demonstrates the value of workforce diversity, and intersectionality theory reveals the complex challenges different groups face. This theoretical foundation helps us understand both achievements and remaining obstacles in creating a more diverse healthcare workforce.

## Appendix D: Graphical illustration of changes over time in the percentage of Arabs in selected health professions

See Fig. 8.

## Appendix E: Place of professional studies

Historically, several of the health professions in Israel have relied heavily on professional studies in institutions of higher learning located in other countries [42]. As a result, as of 2022, among all Israeli health professionals up to age 67, only 42% of physicians, 50% of dentists, and 49% of pharmacists had studied in Israel (Fig. 9); no comparable figure is available for nursing.

For 2012, the percentage of professionals up to age 67 who had studied in Israel was 40% for physicians, 28% for dentists and 52% for pharmacists (the full breakdown is not available for 2012). Thus, between 2012 and 2022 the rate was stable for physicians and pharmacists, while it increased markedly for dentists.

Figure 10 presents data on place of studies for professionals newly licensed in 2020–2022.^35^ The percentage who trained in Israel was 36% for physicians^36^ (down from 48% in 2010), 22% for dentists (down from 31% in 2010), 81% for nurses (no data available for 2010), and 42% for pharmacists (down from 53% in 2010).

For newly licensed physicians, the years between 2010 and 2020–2022 were also characterized by a decline in the share of immigrants who had trained abroad prior to moving to Israel, and conversely a particularly large increase in the share of non-immigrant citizens who trained abroad.

As indicated in Fig. 11, among physicians the high share of non-immigrants who studied abroad is a relatively new phenomenon, compared with dentists and pharmacists. That share grew from approximately 10% in 1995 to slightly more than 25% in 2010 and then to over 50% in 2020.

## Appendix F: Linking stocks and flows—the case of physicians

Table 3 relates the changes in stocks (licensed physicians to age 67) to inflows (new licensees) and outflows (deaths, emigration, and passage of age 67).

**Table 3:** Linking stocks and flows

	Total	Arabs	Jews	Pct Arab
Stock in 2010	25,797	2,049	23,748	8%
Stock in 2022	33,558	6,691	26,867	20%
Change	7,761	4,642	3,119	60%
Inflow	17,299	6,601	10,698	38%
Implied outflow	− 9,538	− 1,959	− 7,579	21%
Pct change	30%	227%	13%	
Pct inflow	67%	322%	45%	
Pct outflow	− 37%	− 96%	− 32%	

As can be seen in that table, in 2010, Israel had 25,797 licensed physicians up to age 67, of whom 2,049 (8%) were Arab and 23,748 (92%) were Jews. In 2022, Israel had 33,558 licensed physicians up to age 67 of whom 6,691 (20%) were Arab and 26,867 (80%) were Jews. Thus, between 2010 and 2022 the number of licensed physicians up to age 67 had increased by 7,761—4,642 among Arabs and 3,119 among Jews.

During this same period, 17,299 new licenses were issued—6,601 to Arabs and 10,698 to Jews. This implies that during the 2020–2022 period the number of physicians who passed away, moved away, or passed the age of 67 was 9,538: 1,959 of them Arabs and 7,579 of them Jews.

## Appendix G: The 2019 Yatziv reform

In January 2023, Israel’s Ministry of Health published a report about its efforts to increase the number of new entrants into the Israeli medical workforce by increasing the size of Israeli medical schools, streamlining the immigration and licensing bureaucracy for new immigrants who are physicians, and providing financial assistance to Israelis accepted into recognized medical schools abroad [51]. That report also contains information about the Yatziv Reform, and a translation of an excerpt from that section of the report appears below:

In 2017, the Licensing Division began to receive reports that the interns coming from certain places abroad lack basic training, and in particular clinical training. A comprehensive examination conducted by Prof. Shaul Yatziv from the Licensing Division revealed that in some foreign medical schools the level of education is insufficient, and they do not provide adequate (sometimes not even any) bedside training (clinical training). Accordingly, a very low percentage of those studying at these universities succeed in passing the licensing exam. Those who do pass the exam are apparently the most prominent among the students from those medical schools, and the low level of training they received is still evident later on.

As a result, the licensing department led the way to a 2019 regulation that stated that students who will begin medical studies in certain schools in 2020 and beyond, will not be able to take the licensing exam in Israel. As a result, new students from Israel have abandoned these universities altogether as of 2020, only a minority of them have moved to other universities abroad and the majority have not started studies at all.

The regulation, known as the Yatziv reform, will, on the one hand, lead to a significant increase in the quality of doctors in Israel and, on the other hand, lead to a drop in the number of doctors entering Israel. The countries where most students study in institutions that are not recognized by the Yatziv reform are: Romania, Ukraine, Moldova, Russia, Armenia, and Egypt.

As can be seen in Fig. 12, in the years leading up the Yatziv reform there was a big increase in students at medical schools which lost their recognition by Israel in that reform. This is consistent with the finding of a recent inter-ministerial committee [15] that “In the period from 2010 to 2021, the number of new licensees in medicine who had studied abroad increased fivefold, with most of the increase occurring in the number of graduates from institutions in Eastern Europe and the Palestinian Authority”.

## Abbreviations

CBS: Central Bureau of Statistics
CHE: Council for Higher Education
MOH: Ministry of Health
IDF: Israel defense forces

## Footnotes

1 One interesting exception is a two-country study involving the US and the UK [18].

2 At the same time, within both the Jewish and the Arab populations there is substantial variation in these and other socioeconomic parameters.

3 The law schools at Tel Avivi University and Bar Ilan University have established programs to assist Israeli Arabs pursuing careers in law. In addition, as a result of a deliberate government effort, the number of Arab lawyers in the public service is substantially greater than it was a decade ago. Progress is still needed at the large private firms.

4 In 2022, the number of licensees up to age 67 was 63,611 for nursing, 33,558 for medicine, 10,660 for dentistry, and 8,947 for pharmacy.

5 Other large health care professions in which Arabs constitute a relatively low proportion of the licensed professionals are physical therapy, occupational therapy, and communication therapy.

6 For two interesting and important exceptions see Popper-Giveon et al. [45] and Reznik [14].

7 The largest incentives are provided for studies in the following departments: economics, psychology, social work, communications disorders, mathematics, computer science, physical natural sciences, chemistry, agriculture, veterinary science, engineering, art and design, and science education.

8 These included Nour Abed Elhadi Shahbari, Alexey Belinsky, Shirli Luz, Eyal Schwartzberg, and Shlomo Zusman.

9 The academic professions span the areas of science and engineering; health care; welfare; business; information and communications technologies; and law, society, and culture.

10 Author's calculation using the Central Bureau of Statistics' 2022 Labor Force Survey.

11 In 2012, the share of Israeli Arabs in employment in all the academic professions taken together was 9% and their share in employment overall was 12%.

12 The changes are clearly quite large, particularly given the limited time period. Contributors include an increase in the percentage of Arabs among new licensees, the aging out of Jewish professionals who immigrated to Israel from FSU countries around 1990, and the large growth in the size of some of the professions as indicated in Fig. 4.

13 The number of newly licensed nurses grew steadily during most of the period and peaked in 2020.

14 Comparable data are not available for nursing. Experts assess that while a non-negligible proportion of nurses studied abroad, that proportion is substantially lower than for physicians, dentists, and pharmacists.

15 For comparative purposes we note that in 2022 Arabs constituted 29% of Israelis aged 20–24.

16 Further details on this can be found in Supplementary Table 2, which includes absolute numbers as well as percents and which distinguishes between health-related departments and other departments.

17 The departments included in “health professions” are medicine, nursing, pharmacy, dentistry, optometry, communications disorders, and allied health professions.

18 The quality of the training varies widely across non-Israeli institutions, from the excellent to the insufficient. Professionals training in high quality institutions outside of Israel can face challenges in securing entry level/residency positions and in securing permanent positions in sought after institutions. Initially there can also be adjustment challenges due to differences in medical cultures. There are cases of brain drain, but this has not surfaced as a major problem to date.

19 In 2022, the jurisdictions aside from Israel in which the most newly licensed Israeli-born professionals had studied, were as follows: for medicine—Romania (288), Moldova (160), Ukraine (112), the Palestinian Authority (59), Hungary (53), and Jordan (53). For dentistry—the Palestinian Authority (114), Romania (46), Ukraine (43), and Moldova (35); for pharmacy—the Palestinian Authority (59) and Jordan (31); for nursing—the Palestinian Authority (528) and Jordan (43). The numbers in parentheses are the number of newly licensed professionals who had studied in each jurisdiction.

20 There are no internationally recognized and quantified standards for adequate or optimal minority representation in health care professions. In the United States, the proportion of Blacks and Hispanics in key health professions are considered problematic as they are well below the proportion of those groups in the general population. With regard to the four health professions studied in this paper, the situation in Israel is clearly much better, as the percentage of Arabs in these professions is equal to or exceed their percentage in the general population.

21 Periods of conflict, such as the recent Israel-Hamas war, exacerbate tensions between Arab and Jewish healthcare professionals. In mixed teams, political tensions can affect collaboration, trust, and communication [41]. Instances of intergroup conflict in hospitals during heightened political crises have required interventions, including diversity and conflict-resolution training [43].

22 There is a chicken and egg issue here. One of the reasons that Israel historically did not expand its professional training capacity was the ready availability of large numbers of olim who trained abroad before moving to Israel.

23 The peace agreements of the mid-1990s with Jordan and the Palestinian Authority expanded opportunities for Arab students to study healthcare professions abroad.

24 This statement refers to licensed professionals of all ages. A recent study by Burger et al. has found that among newly licensed physicians, it takes Arabs longer to secure residency positions [67].

25 Similarly, in dentistry there was an increase in the Arab share of new licenses at the same time as a decrease in the Arab share in first-degree students. Here, the reconciling factor might be the increase in the share of new licensees who studied in the Palestinian Authority.

26 Nursing is also unique in that it is the only one of the four professions studied for which professional training programs have been established in colleges that serve primarily Arab students.

27 For example, several of the CEOs of Israel’s major acute care hospitals are Israeli Arabs, but none of the major hospital CEOs studied in professional schools outside of Israel.

28 In the UK, there is currently a major effort underway to increase the representation of Black people and minorities in medical leadership roles [68]. This could be a key area for UK-Israel cross-national learning.

29 As a result, Bedouin living in the south often received their primary care from Arab physicians and nurses from the north who had moved temporarily to the south. Differences in culture and class made this a less-than-ideal situation. This situation has improved as more Bedouin enter the health professions [64].

30 Moving beyond the domain of the health care professions to the related domain of patient care – there continue to be significant Arab–Jewish disparities across many dimensions of health care and health status [33–35]. Concrete steps are underway to address many of these challenges [50], and hopefully we will see real progress in the years ahead.

31 Per the Yatziv reform, starting in 2026 the Israeli physician licensure exam may be taken only by graduates of medical schools located in OECD countries or graduates of other medical schools that are recognized by Western countries (particularly by the U.S.).

32 The Yatziv reform is similar in some ways to the Flexner Reform of US and Canadian medical education at the beginning of the twentieth century [77].

33 The numbers of Israelis completing studies in the medical schools not recognized by the Yatziv reform increased significantly in the five years leading up to the 2019 Yatziv reform. Once the reform went into effect in 2020 almost no Israelis have initiated studies in those medical schools [51].

34 Currently, none of the four health care fields considered in this article are among the fields for which the Council of Higher Education currently offers special financial support to Arab students.
