# Supplementary material for: Arab representation in Israeli healthcare professions: achievements, challenges and opportunities
Source: Isr J Health Policy Res. 2025 Feb 3;14:7. doi: 10.1186/s13584-024-00663-3 (PMC11789336; doi:10.1186/s13584-024-00663-3)
Supplement: Supplementary file 1 — Additional file 1 [file 13584_2024_663_MOESM1_ESM.docx]

**Supplementary Table 1**

**Occupational distributions, 2022**

|  | **Total** | **Jews** | **Arabs** |  | **% Arabs** |
| --- | --- | --- | --- | --- | --- |
|  |  |  |  |  |  |
| **Total** | **100%** | **100%** | **100%** |  | **14%** |
| Managers | 8% | 9% | 4% |  | 7% |
| Professionals | 30% | 33% | 20% |  | 9% |
| Technicians | 14% | 16% | 7% |  | 7% |
| Office workers | 6% | 7% | 5% |  | 11% |
| Sales and service workers | 18% | 16% | 20% |  | 16% |
| Skilled workers in agriculture | 1% | 1% | 1% |  | 19% |
| Skilled workers outside agriculture | 12% | 9% | 30% |  | 35% |
| Unskilled workers | 5% | 3% | 10% |  | 32% |
| Unknown | 7% | 7% | 5% |  | 10% |
|  |  |  |  |  |  |
|  |  |  |  |  |  |
| **All professions** | **30%** | **33%** | **20%** |  | **9%** |
| Science and engineering | 5% | 6% | 2% |  | 6% |
| Health | 4% | 3% | 5% |  | 19% |
| Education | 8% | 8% | 9% |  | 16% |
| Management | 4% | 4% | 1% |  | 5% |
| Information and communication technology | 4% | 6% | 1% |  | 2% |
| Law, society, culture | 5% | 6% | 2% |  | 7% |

Source: CBS Labor Force Survey

**Supplementary Table 2**

**The change in the number of newly licensed professionals**

**(by nationality, 2010 to 2022)**

|  | **Total** | **Arabs** | **Jews** |
| --- | --- | --- | --- |
| 1. **Percent change from 2010 to 2022** |  |  |  |
| Physicians | 168% | 584% | 76% |
| Nurses | 274% | 449% | 199% |
| Dentists | 100% | 120% | 87% |
| Pharmacists | 24% | 14% | 34% |
|  |  |  |  |
| 1. **Absolute change from 2010 to 2022** |  |  |  |
| Physicians | 1,219 | 764 | 455 |
| Nurses | 2,547 | 1,251 | 1,296 |
| Dentists | 267 | 128 | 139 |
| Pharmacists | 58 | 17 | 41 |
|  |  |  |  |
| 1. **Newly licensed in 2022** |  |  |  |
| Physicians | 1,946 | 895 | 1,051 |
| Nurses | 3,476 | 1,529 | 1,947 |
| Dentists | 534 | 235 | 299 |
| Pharmacists | 303 | 139 | 164 |
|  |  |  |  |
| 1. **Newly licensed in 2010** |  |  |  |
| Physicians | 727 | 131 | 596 |
| Nurses | 929 | 279 | 650 |
| Dentists | 267 | 107 | 160 |
| Pharmacists | 245 | 122 | 123 |

Source: Secondary analysis based on data from Ministry of Health (Haklai et al, 2024)

Note that the data on absolute numbers of professionals in the original source do not distinguish between Arabs and Jews. The original source does include data on the total absolute number of professionals and the percentage of these who are Arabs and Jews. We used those two parameters to calculate some of the numbers presented in this table.

**Supplementary Table 3**

| **Distribution of first-degree students in Israeli universities,  by departmental clusters** | | | | | |
| --- | --- | --- | --- | --- | --- |
| Comparison of all students and Arab students, for 2012/3 and 2022/3 | | | | | |
|  |  |  |  |  |  |
|  | **All students** | |  | **Arab students** | |
|  | **2012-3** | **2022-3** |  | **2012-3** | **2022-3** |
|  |  |  |  |  |  |
| **Total** | **100%** | **100%** |  | **100%** | **100%** |
| Humanities | 17% | 9% |  | 18% | 10% |
| Education | 2% | 1% |  | 3% | 2% |
| Social sciences | 26% | 20% |  | 31% | 13% |
| Management and law | 7% | 11% |  | 4% | 9% |
| Health professions | 11% | 11% |  | 20% | 16% |
| Natural sciences | 17% | 23% |  | 14% | 24% |
| Engineering and architecture | 20% | 24% |  | 11% | 27% |

Source: Analysis of data from the Council for Higher education

**Supplementary Table 4**

| **Women as a percent of employees,** | | |
| --- | --- | --- |
| **in selected health professions** | | |
|  |  |  |
| **A. Total** | **2010** | **2023** |
|  |  |  |
| Medicine | 46 | 43 |
| Dentistry | 34 | 42 |
| Nursing | 86 | 84 |
| Pharmacy | NA | 69 |
|  |  |  |
| **B. Arabs** | **2010** | **2023** |
|  |  |  |
| Medicine | 17 | 22 |
| Dentistry | 5 | 38 |
| Nursing | 53 | 64 |
| Pharmacy | NA | 53 |
|  |  |  |
| **C. Jews** | **2010** | **2023** |
|  |  |  |
| Medicine | 48 | 51 |
| Dentistry | 41 | 43 |
| Nursing | 93 | 92 |
| Pharmacy | NA | 84 |

**Supplementary Table 5**

| **Summary chart - Arab representation in key groups at two points in time** | | | |  |
| --- | --- | --- | --- | --- |
|  |  |  |  |  |
| **2010 or 2012** | Physicians | Dentists | Nurses | Pharmacists |
|  |  |  |  |  |
| Working professionals | 8 | 19 | 17 | 28 |
| Licensed professionals up to age 67 | 9 | 17 | 12 | 33 |
|  |  |  |  |  |
| Newly licensed professionals | 18 | 40 | 32 | 50 |
| First degree students in universities | 18 | 38 | 37 | 41 |
|  |  |  |  |  |
|  |  |  |  |  |
| **2022 or 2023** | Physicians | Dentists | Nurses | Pharmacists |
|  |  |  |  |  |
| Working professionals | 25 | 27 | 27 | 49 |
| Licensed professionals up to age 67 | 25 | 36 | 27 | 49 |
|  |  |  |  |  |
| Newly licensed professionals | 46 | 44 | 44 | 46 |
| First degree students in universities | 9 | 23 | 33 | 70 |
